# Dual transcriptome analysis reveals differential gene expression modulation influenced by *Leishmania* arginase and host genetic background

**DOI:** 10.1099/mgen.0.000427

**Published:** 2020-09-04

**Authors:** Juliana Ide Aoki, Sandra Marcia Muxel, Maria Fernanda Laranjeira-Silva, Ricardo Andrade Zampieri, Karl Erik Müller, Audun Helge Nerland, Lucile Maria Floeter-Winter

**Affiliations:** ^1^​ Department of Physiology, Institute of Bioscience, University of São Paulo, São Paulo, Brazil; ^2^​ Department of Clinical Science, University of Bergen, Bergen, Norway; ^3^​ Department of Internal Medicine, Drammen Hospital, Drammen, Norway

**Keywords:** arginine transport, BALB/c, C57BL/6, immune response, *Leishmania amazonensis*, macrophage infection, RNA-seq

## Abstract

The outcome of *Leishmania* infection is strongly influenced by the host’s genetic background. BALB/c mice are susceptible to *Leishmania* infection, while C57BL/6 mice show discrete resistance. Central to the fate of the infection is the availability of l-arginine and the related metabolic processes in the host and parasite. Depending on l-arginine availability, nitric oxide synthase 2 (NOS2) of the host cell produces nitric oxide (NO) controlling the parasite growth. On the other hand, *Leishmania* can also use host l-arginine for the production of polyamines through its own arginase activity, thus favouring parasite replication. Considering RNA-seq data, we analysed the dual modulation of host and parasite gene expression of BALB/c or C57BL/6 mouse bone marrow-derived macrophages (BMDMs) after 4 h of infection with *Leishmania amazonensis* wild-type (*La*-WT) or *L. amazonensis* arginase knockout (*La*-arg^-^). We identified 12 641 host transcripts and 8282 parasite transcripts by alignment analysis with the respective *Mus musculus* and *L. mexicana* genomes. The comparison of BALB/c_*La*-arg^-^ *versus* BALB/c_*La*-WT revealed 233 modulated transcripts, with most related to the immune response and some related to the amino acid transporters and l-arginine metabolism. In contrast, the comparison of C57BL/6_*La*-arg^-^ *vs.* C57BL/6_*La*-WT revealed only 30 modulated transcripts, including some related to the immune response but none related to amino acid transport or l-arginine metabolism. The transcriptome profiles of the intracellular amastigote revealed 94 modulated transcripts in the comparison of *La*-arg^-^_BALB/c *vs. La*-WT_BALB/c and 45 modulated transcripts in the comparison of *La*-arg^-^_C57BL/6 *vs. La*-WT_C57BL/6. Taken together, our data present new insights into the impact of parasite arginase activity on the orchestration of the host gene expression modulation, including in the immune response and amino acid transport and metabolism, mainly in susceptible BALB/c-infected macrophages. Moreover, we show how parasite arginase activity affects parasite gene expression modulation, including amino acid uptake and amastin expression.

## Data Summary

The authors confirm all supporting data, code and protocols have been provided within the article and through supplementary data files. Sequencing data are available in the NCBI BioProject database (https://www.ncbi.nlm.nih.gov/bioproject/) under accession numbers PRJNA481041 and PRJNA481042 and in the Sequence Read Archive (SRA) database (https://www.ncbi.nlm.nih.gov/sra) under accession numbers SRP156183 and SRP156466.

**Impact Statement**

RNA-seq methodology has improved the knowledge of *Leishmania* infection. In this work, we describe the transcriptome profiles of BALB/c and C57BL/6 mouse macrophages infected with *L. amazonensis* wild-type or *L. amazonensis* arginase knockout. The analyses show that the activity of the parasite arginase affects gene expression modulation to evade the host immune response and enable parasite survival inside macrophages in a host genetic background-dependent manner.

## Introduction

Leishmaniases are a group of diseases caused by digenetic protozoan parasites of the genus *Leishmania*, which are transmitted by sand flies of the subfamily Phebotominae. These diseases are characterized by cutaneous, mucosal and/or visceral manifestations. Based on recent estimates, 0.7–1 million new cases of leishmaniases are reported annually in nearly 100 endemic countries [1].

*Leishmania* infection can result in different outcomes depending on the host immune response and the parasite species [2–8]. As an obligate intracellular parasite, *Leishmania* modulates the immune response to avoid the control of the infection by the host [2, 9–12]. The parasite life cycle differs in the sand-fly vector and the mammalian host due to differences in temperature, pH and nutritional availability, leading to dynamic modifications in cellular signalling, gene expression, morphology and metabolism [13].

The polyamines pathway is highly regulated at multiple levels, including transcriptional, translational and post-translational, highlighting the important role of this pathway in cell growth regulation. During *Leishmania* infection, depending on the availability of l-arginine in the host, nitric oxide (NO) is produced by nitric oxide synthase 2 (NOS2), which can lead to parasite killing. On the other hand, host and parasite arginase expression triggers polyamines production, which can lead to parasite replication [2, 10, 12].

The importance of the polyamines pathway in *Leishmania* has been described with mutants that do not express essential enzymes involved in this pathway, such as arginase, ornithine decarboxylase or spermidine synthase [14–19]. As a result, parasite arginase has been described as an essential player in the polyamines pathway of *L. amazonensis*, impacting parasite replication and infectivity [14, 20, 21]. Additionally, the role of arginase has also been related to virulence factors, such as glycoprotein 63, lipophosphoglycan, proteophosphoglycan, cysteine peptidase, autophagy genes, superoxide dismutases, ascorbate peroxidase and amastins [22].

RNA-seq technology has been used to describe transcriptome profiles of both host and parasite, providing additional knowledge about *Leishmania* biology and the coordination of host immune responses to infection [23–30]. In this work, we describe gene expression modulation based on dual transcriptome profiles of early *in vitro La*-WT and *La*-arg^-^ infections of bone marrow-derived macrophages (BMDMs) from BALB/c or C57BL/6 mice. Using RNA-seq methodology, we identified 12 641 host transcripts through alignment to the *M. musculus* genome and 8282 parasite transcripts through alignment to the *L. mexicana* genome.

In a recent work from our group, we focused on the differential modulation of the immune response to *La*-WT infection of hosts with different genetic backgrounds [9]. In the present work, we show how the parasite arginase activity impacts on the modulation of the host immune response and l-arginine uptake and metabolism at the beginning of *in vitro* infection and how the host genetic background can influence the infection outcome.

## Methods

### Animals

Female BALB/c and C57BL/6 mice (6–8 weeks old) (five animals per group) were obtained from the Animal Centre of the Medical School of the University of São Paulo and maintained at the Animal Centre of the Department of Physiology at the Institute of Bioscience of the University of São Paulo, receiving food and water *ad libitum*.

### Ethics statement

The protocols for the animals experiments were approved by the Animal Care and Use Committee at the Institute of Bioscience of the University of São Paulo (CEUA 233/2015). This study was conducted based on the recommendations and the policies for the Care and Use of Laboratory Animals of São Paulo State (State Law 11.977, from 25 August 2005) and the Brazilian government (Federal Law 11.794, from 8 October 2008).

### *Leishmania* culture

Promastigotes of *L. amazonensis* (MHOM/BR/1973/M2269) wild-type (*La*-WT) and *L. amazonensis* arginase knockout (*La*-arg^-^) [31] were grown at 25 °C in M199 medium (Gibco), pH 7.0, supplemented with l-glutamine, 10 % heat-inactivated fetal bovine serum, 0.25 % hemin, 40 mM NaHCO_3_, 100 µM adenine, 40 mM HEPES, 100 U ml^−1^ penicillin and 100 μg ml^−1^ streptomycin. For the *La*-arg^-^ cultures, hygromycin (30 μg ml^−1^), puromycin (30 μg ml^−1^) and putrescine (50 µM) were added to the medium. The parasites were counted in a Neubauer chamber.

### *In vitro* macrophage infection

BMDMs were differentiated from cells obtained from BALB/c or C57BL/6 mice. The cells were collected from femurs that had been washed with PBS and centrifuged at 500 ***g*** for 10 min at 4 °C, and erythrocytes were lysed by the addition of NH_4_Cl (145 mM) and Tris-base (200 mM) pH 7.0, followed by incubation on ice for 20 min. Then, the cells were washed with cold PBS, centrifuged at 500 ***g*** for 10 min at 4 °C and incubated in RPMI 1640 medium supplemented with penicillin (100 U ml^−1^), streptomycin (100 µg ml^−1^), 2-mercaptoethanol (50 µM), l-glutamine (2 mM), sodium pyruvate (1 mM), 10 % fetal bovine serum and 10 % L929 conditioned medium as the source of macrophage stimulation factors. The cells were differentiated for 7 days at 34 °C in 5 % CO_2_. BMDMs were used after phenotypic flow cytometry analysis, showing a population of at least 95 % F4/80 and CD11b-positive cells, as previously described [9, 32]. After macrophage differentiation, cellular viability was evaluated with Trypan blue staining, and the cells were counted in a Neubauer chamber.

Approximately 5×10^6^ BMDMs from BALB/c or C57BL/6 mice were incubated overnight in sterile six-well plates (SPL) at 34 °C and 5 % CO_2_. Non-adherent cells were removed by washing with PBS. *La*-WT or *La*-arg^-^ promastigotes at the stationary growth phase were inoculated into the wells at a ratio of five parasites per one macrophage (MOI 5 : 1). After 4 h of infection, the cells were washed with PBS, RNA was isolated and the infection index was determined. Non-infected macrophages maintained in culture under the same conditions were used as the controls. The infection was evaluated by determining the percentage of infected cells in 400 panoptic-stained (Laborclin) macrophages. The infection index was determined by multiplying the percentage of infected macrophages and the mean number of parasites per infected cell [33, 34].

### Total RNA isolation and library construction

Total RNA from five independent biological replicates was isolated using TRIzol reagent (Life Technologies) according to the manufacturer’s instructions. RNA samples were treated with DNase I (1 U per 1 µg of RNA) (Thermo Scientific) at 37 °C for 1 h, and the RNA concentration was determined using a spectrophotometer Nanodrop ND1000 spectrophotometer (Thermo Scientific). In addition, RNA integrity was evaluated using an Agilent 2100 Bioanalyzer and a Pico Agilent RNA 6000 kit (Agilent Technologies) according to the manufacturers’ instructions. rRNA depletion was performed using a poly (A) magnetic beads capture protocol and a TrueSeq Stranded Total RNA Sample Prep kit (Illumina) according to the manufacturers’ instructions. Libraries were prepared using a TrueSeq Stranded RNA-seq Library Prep kit (Illumina), according to the manufacturers’ instructions.

### RNA-seq and data analysis

Paired-end reads (100 bp) were obtained using the Illumina NovaSeq 6000 platform at Macrogen (Seoul, South Korea). Quality control was performed on the sequenced raw reads based on read quality, total bases, total reads, GC (%) and basic statistics. The quality of the reads was also analysed using FastQC according to the Phred quality score [35]. Reads with Phred quality scores lower than 20 were discarded. To reduce bias in the analysis and the number of artefacts, such as low-quality reads and adaptor sequences, the Trimmomatic programme was used [36]. Trimmed reads were mapped to the *L. mexicana* reference genome (MHOMGT2001U1103) based on the genomic data obtained from TriTrypDB version 36 (https://tritrypdb.org/tritrypdb/app) using the TopHat splice-aware aligner [37, 38]. A maximum of two mismatches were allowed. The transcripts were assembled from the aligned reads by Cufflinks, providing information on known transcripts. The expression profiles of the assembled transcripts and abundance estimation for each sample were also generated by Cufflinks [39]. The expression profiles were calculated as fragments per kilobase of transcript per million mapped reads (FPKM). These data are presented as normalized values based on the transcript length and coverage depth [40]. Gene expression level values were calculated from the transcript counts. DEG analysis was performed based on the following cell comparisons: (1) BALB/c_*La*-arg^-^ *vs.* BALB/c non-infected; (2) BALB/c_*La*-arg^-^ *vs.* BALB/c_*La*-WT; (3) BALB/c_*La*-arg^-^ *vs.* C57BL/6_*La*-arg^-^; (4) C57BL/6_*La*-arg^-^ *vs.* C57BL/6 non-infected; (5) C57BL/6_*La*-arg^-^ *vs.* C57BL/6_*La*-WT. Genes with a zero FPKM value were excluded. Groups treated under different conditions or with differentially expressed genes were filtered out through statistical hypothesis tests. The false discovery rate (FDR) was controlled by adjusting the *p*-value using the Benjamini–Hochberg algorithm [41]. Functional annotation was performed using Gene Ontology (GO) and Kyoto Encyclopedia of Genes and Genomes (KEGG) analyses. All analyses were performed by Macrogen (Seoul, South Korea).

### RT-qPCR validation assays

Reverse transcriptase quantitative PCR (RT-qPCR) validation assays were performed for five biological replicates using 2 µg of total RNA as the template, reverse transcriptase (Thermo-Scientific) and random primers (Thermo-Scientific), according to the manufacturer's instructions. Equal amounts of cDNA were assessed in a total volume of 25 µl containing Power SYBR Green qPCR master mix (Life Technologies) and the primers (200 nM) (Table S1, available in the online version of this article). The mixture was incubated at 94 °C for 5 min, followed by 40 cycles at 94 °C for 30 s, 60 °C for 30 s and 72 °C for 30 s. A negative control without reverse transcriptase was included to detect genomic DNA contamination in the cDNA samples. Reactions were carried out using a PikoReal real-time PCR system (Thermo Scientific). Reactions were performed in duplicate and analyses were performed using PikoReal Software 2.2 (Thermo Scientific). The copy number of the target and the reference gene were quantified, considering the molar-mass concentration, according to a standard curve generated from a tenfold serial dilution of a quantified PCR product. The normalized *target*/*gapdh* ratio of the absolute number of each target is reported as the expression level.

### *In silico* analysis

Network mapping based on a co-expression analysis was performed using the GeneMANIA prediction server [42].

### Statistical analysis

The experiments were performed with five biological replicates per group. The results are presented as the mean±standard deviation. A DEG profile was considered statistically significant considering fold change ≥2, *p*-value <0.05 and FDR analysis. RT-qPCR validation assays were considered statistically significant based on Student’s *t*-test and with *p*-value <0.05.

## Results and discussion

### Parasite arginase does not impact the infection index of BALB/c or C57BL/6 macrophages during early infection

Experimental murine infections with *L. amazonensis* have demonstrated distinct susceptibility compared to *L. major* infection [3, 43–45]. *L. amazonensis* induces severe lesions in susceptible BALB/c mice but causes moderate lesions in resistant C57BL/6 mice [6]. The differences in the infection progression among distinct mouse strains include lesion size, parasite burden and immune response [6, 44]. Furthermore, we have recently published a transcriptomic data analysis showing differences in the modulation of gene expression in BALB/c and C57BL/6 in response to early *in vitro L. amazonensis* infection, highlighting that host genetic background defines the outcome of infection [9].

In the present work, macrophages from the BALB/c and C57BL/6 mouse strains were infected with *La*-WT or *La*-arg^-^ (MOI 5 : 1), and the infection indexes were analysed after 4 h. No significant differences in infectivity were observed for BALB/c_*La*-arg^-^ compared to BALB/c_*La*-WT or for C57BL/6_*La*-arg^-^ compared to C57BL/6_*La*-WT (Fig. S1). This profile corroborates previous findings that parasite arginase does not impact the parasite entry at the beginning of infection [21, 31, 32]. A reduction in the infection index in both *La*-arg^-^-infected BALB/c and C57BL/6 macrophages was observed only after 48 and 72 h [21, 31, 32].

### Parasite arginase activity differentially modulates the gene expression levels of BALB/c and C57BL/6 macrophages

Although the infection indexes were not altered after 4 h of infection, most gene expression modulation has been described as occurring during the time of initial infection [23, 24, 32, 46], indicating a coordinated gene expression modulation that may later favour parasite survival in the host macrophage, depending on the host genetic background. Transcriptome profiles were obtained through Illumina sequencing and alignment with the *M. musculus* reference genome. According to this analysis, we identified 12 641 total transcripts (Table S2), and only 17 % transcripts were differentially expressed, considering fold change ≥2 and *p*-value <0.05. Host gene expression modulation in response to *La*-WT infection was previously assessed by our group and recently published [9].

**Fig. 1.**
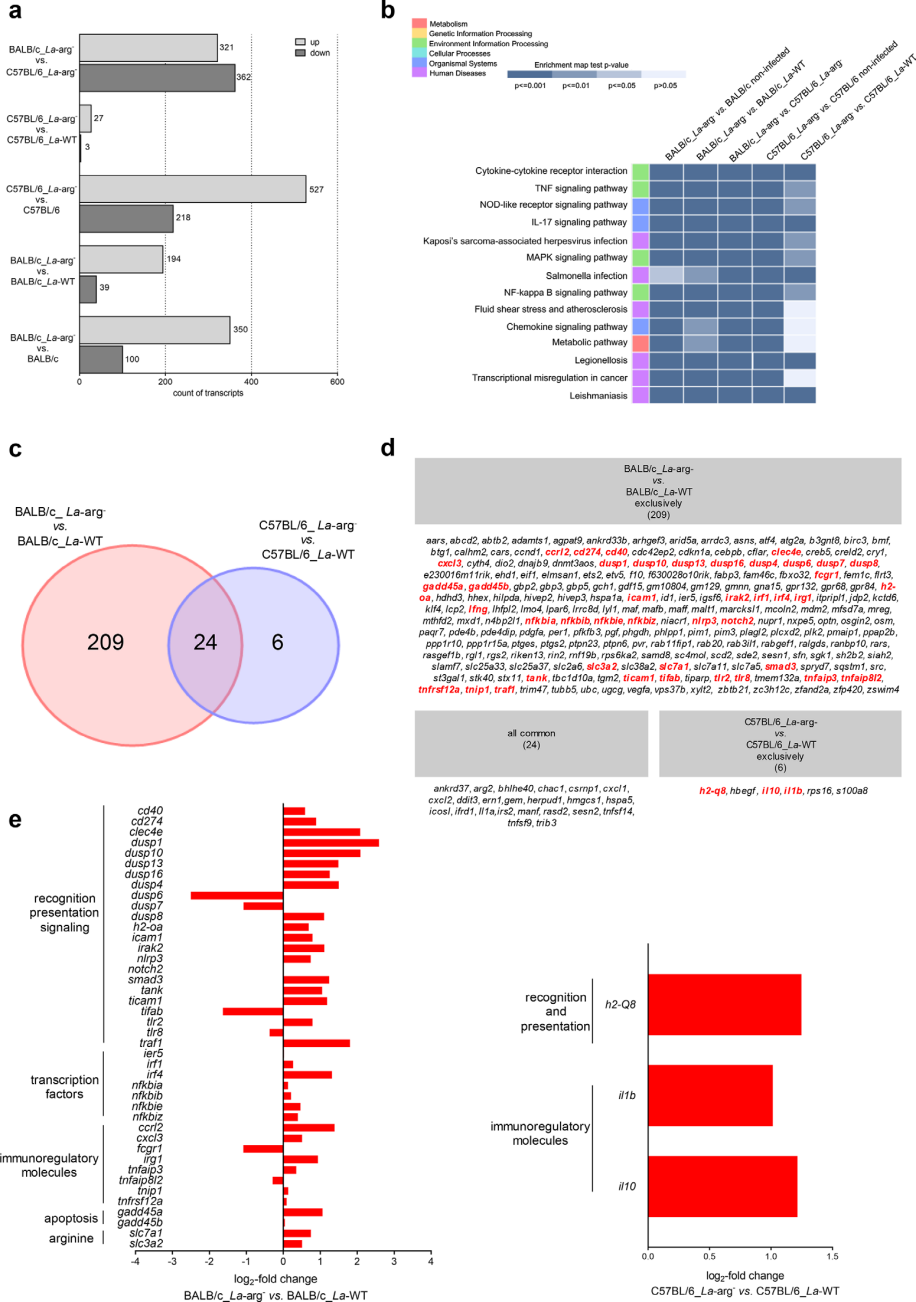
Functional analysis of the differentially expressed gene profiles of BALB/c and C57BL/6 macrophages infected with *La*-WT or *La*-arg^-^ for 4 h. (a) Number of upregulated (light grey) and downregulated (dark grey) differentially expressed genes. (b) KEGG enrichment analysis shown as a heat map of the 14 most regulated pathways based on the indicated comparisons. (c) Venn diagram of the 263 DEGs based on the comparisons of BALB/c_*La*-arg^-^ *vs.* BALB/c_*La*-WT and C57BL/6_*La*-arg^-^ *vs.* C57BL/6_*La*-WT. (d) List of the exclusively and commonly transcripts for each comparison according to the Venn diagram analysis. The red transcripts are related to the immune response, and arginine transport and metabolism. (e) DEG profiles as indicated by log_2_-fold changes of the exclusively modulated genes involved in the immune response and arginine transport and metabolism in the comparisons of BALB/c_*La*-arg^-^ *vs.* BALB/c_*La*-WT and C57BL/6_*La*-arg^-^ *vs.* C57BL/6_*La*-WT. *L. amazonensis* wild-type (*La*-WT) and *L. amazonensis* arginase knockout (*La*-arg^-^). The data are from five independent biological replicates, considering fold change ≥2 and a *p*-value <0.05.

For the study presented here, we evaluated how the parasite arginase activity impacts host gene expression modulation. From the comparison of BALB/c_*La*-arg^-^ *vs.* BALB/c_*La*-WT, we identified 39 downregulated and 194 upregulated transcripts. In contrast, from the comparison of C57BL/6_*La*-arg^-^ *vs.* C57BL/6_*La*-WT, we identified only 3 downregulated and 27 upregulated transcripts. Additionally, from the comparison of BALB/c_ *La*-arg^-^ *vs.* BALB/c non-infected, we identified 100 downregulated and 350 upregulated transcripts; and from the comparison of C57BL/6_ *La*-arg^-^ *vs.* C57BL/6 non-infected, we identified 218 downregulated and 527 upregulated transcripts (Fig. 1a).

**Fig. 2.**
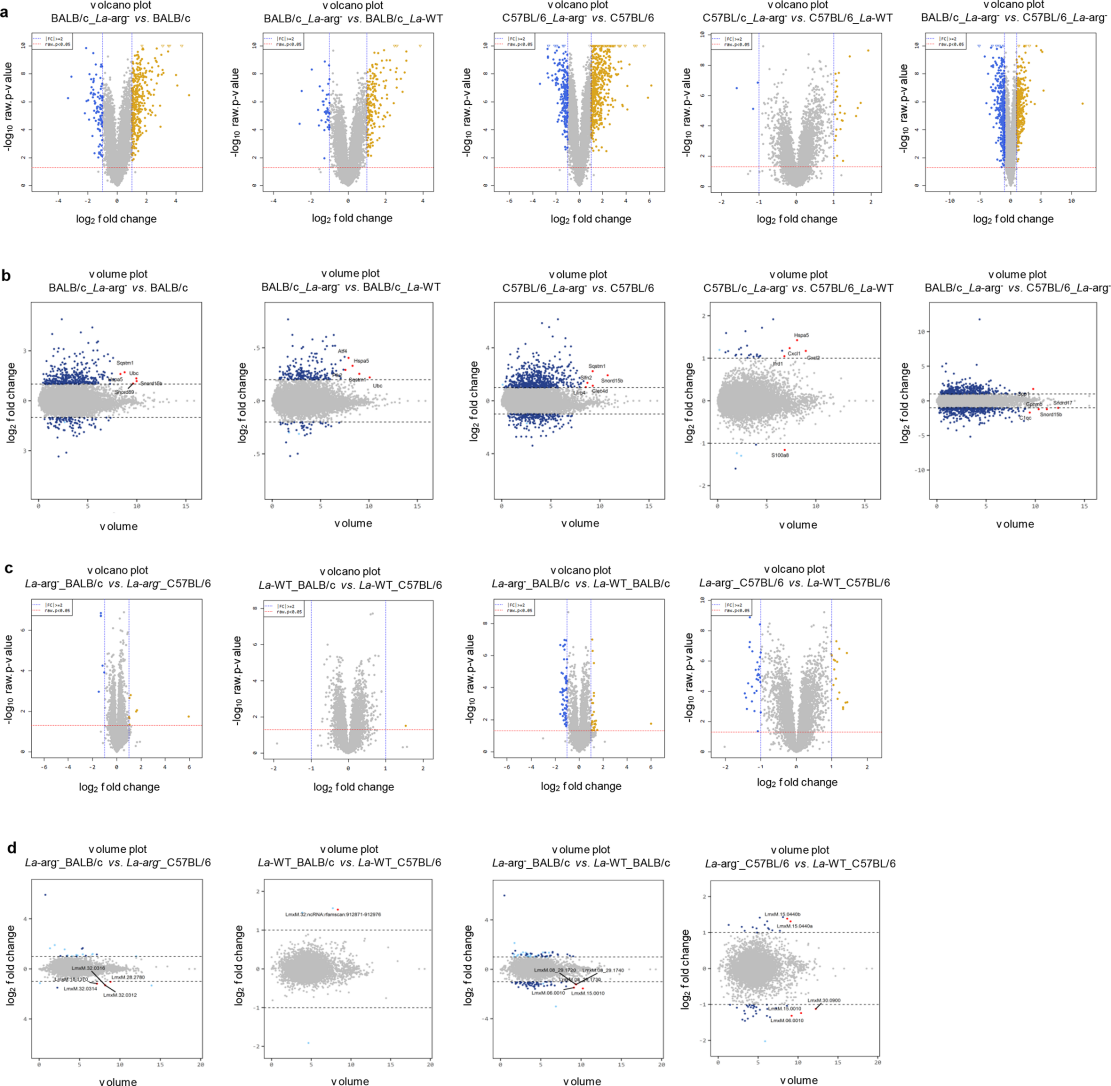
Fold-change expression profiles of the differentially expressed genes in BALB/c and C57BL/6 macrophages infected with *La*-WT or *La*-arg^-^ and in *La*-WT and *La*-arg^-^ intracellular amastigotes in BALB/c and C57BL/6 macrophages after 4 h of infection. (a) Volcano plots of the comparisons of BALB/c and C57BL/6 macrophages infected with *La*-WT or *La*-arg^-^, considering genes with a fold change ≥2 and a *p*-value <0.05. Genes significantly upregulated (yellow dots) are in the upper right square of each graph (positive log-fold-change value). Genes significantly downregulated (blue dots) are in the upper left square of each graph (negative log-fold-change value). (b) Volume plots of the comparisons of BALB/c and C57BL/6 macrophages infected with *La*-WT or *La*-arg^-^, based on genes with log_2_-fold change ≥2 and a *p*-value <0.05 (blue dots). The top five significantly upregulated and downregulated genes are represented by red dots. (c) Volcano plots of the comparisons of *La*-WT and *La*-arg^-^ intracellular amastigotes in BALB/c and C57BL/6 macrophages, considering genes with fold change ≥2 and a *p*-value <0.05. Genes significantly upregulated (yellow dots) are in the upper right square of each graph (positive log-fold-change value). Genes significantly downregulated (blue dots) are in the upper left square of each graph (negative log-fold-change value). (d) Volume plots of the comparisons of *La*-WT and *La*-arg^-^ intracellular amastigotes in BALB/c and C57BL/6 macrophages, considering genes with log_2_-fold change ≥2 and a *p*-value <0.05 (blue dots). The top five significantly upregulated and downregulated genes are represented by red dots. *L. amazonensis* wild-type (*La*-WT) and *L. amazonensis* arginase knockout (*La*-arg^-^).

Based on the DEG profiles, we generated volcano plots comparing the expression fold change (log_2_) with the corresponding adjusted *p*-value (-log_10_) (Fig. 2a). We further analysed the volume plot (Fig. 2b) and identified the top five regulated transcripts for each comparison, showing a modulation of transcripts involved in the synthesis and regulation of ribosome activity, as well as RNA folding and genes related to the immune response (Table 1).

**Table 1.** Top five differentially expressed genes in the BALB/c and C57BL/6 macrophages infected with *La*-WT or *La*-arg^-^ for 4 h

ID	Product description	Fold change	*p*-value
BALB/c_*La*-arg- *vs.* BALB/c non-infected
*sqstm1*	*sequestosome-1 isoform 2*	3.27	1.84^−10^
*ubc*	*ubiquitin C*	2.53	4.92^−05^
*hspa5*	*heat-shock protein 5*	3.04	3.28^−06^
*snord15b*	*small nucleolar RNA, C/D box 14B*	2.26	0.002
*snord89*	*small nucleolar RNA, C/D box 89*	2.04	0.015
	BALB/c_*La*-arg^-^ *vs*. BALB/c_*La*-WT		
*atf4*	*activating transcription factor 4*	4.06	3.04^−08^
*hspa5*	*heat-shock protein 5*	3.14	6.25^−08^
*plk2*	*polo-like kinase 2*	2.74	1.45^−08^
*sqstm1*	*sequestosome-1 isoform 2*	2.41	2.65^−09^
*ubc*	*ubiquitin C*	2.15	2.22^−08^
	C57BL/6_*La*-arg^-^ *vs.* C57BL/6 non-infected		
*sqstm1*	*sequestosome-1 isoform 2*	4.69	1.83^−09^
*slfn2*	*schlafen 2*	2.55	4.39^−11^
*snord15b*	*small nucleolar RNA, C/D box 14B*	3.79	1.79^−06^
*lilrb4*	*leucocyte immunoglobulin-like receptor subfamily B member 4 isoform 2*	2.03	3.69^−06^
*clec4d*	*c-type lectin domain family 4, member d*	2.19	4.81^−07^
	C57BL/6_*La*-arg^-^ *vs.* C57BL/6_*La*-WT		
*hspa5*	*heat-shock protein 5*	2.68	2.55^−09^
*cxcl1*	*chemokine (C-X-C motif) ligand 1*	2.36	4.53^−05^
*cxcl2*	*chemokine (C-X-C motif) ligand 2*	2.25	1.29^−05^
*ifrd1*	*interferon-related developmental regulator 1*	2.07	3.66^−08^
*s100a8*	*s100 calcium binding protein A8 (calgranulin A*)	−2.23	7.46^−06^
	BALB/c_*La*-arg^-^ *vs.* C57BL/6_*La*-arg^-^		
*spp1*	*secreted phosphoprotein 1*	3.29	1.40^−04^
*gpnmb*	*glycoprotein (transmembrane) nmb*	−2.29	2.90^−07^
*snord17*	*small nucleolar RNA, C/D box 17*	−2.02	2.85^−04^
*snord15b*	*small nucleolar RNA, C/D box 14B*	−2.30	2.2^−03^
*c1qc*	*complement component 1, q subcomponent, C chain*	−3.22	4.84^−08^

The top five most upregulated and downregulated genes among 12 641 transcripts previously defined as DEGs based on the following comparisons: BALB/c_*La*-arg^-^ *vs.* BALB/c non-infected, BALB/c_*La*-arg^-^ *vs.* BALB/c_*La*-WT, C57BL/6_*La*-arg^-^ *vs.* C57BL/6 non-infected, C57BL/6_*La*-arg^-^ *vs.* C57BL/6_*La*-WT and BALB/c_*La*-arg^-^ *vs.* C57BL/6_*La*-arg^-^ *,* based on the criteria of a fold change ≥2 and *p*-value ˂ 0.05. The list is based on the volume plot of differentially expressed genes. *L. amazonensis* wild-type (*La*-WT) and *L. amazonensis* arginase knockout (*La*-arg^-^).

Functional annotation and gene enrichment analysis were performed using the GO and KEGG databases, which showed that the 14 most differentially regulated pathways were mainly related to immune response signalling (Fig. 1b). A Venn diagram analysis was also performed and revealed 209 exclusively modulated genes in BALB/c_*La*-arg^-^ *vs*. BALB/c_*La*-WT comparison, 6 exclusively modulated genes in C57BL/6_*La*-arg^-^ *vs*. C57BL/6_*La*-WT comparison and 24 commonly modulated genes (Fig. 1c, d).

**Fig. 3.**
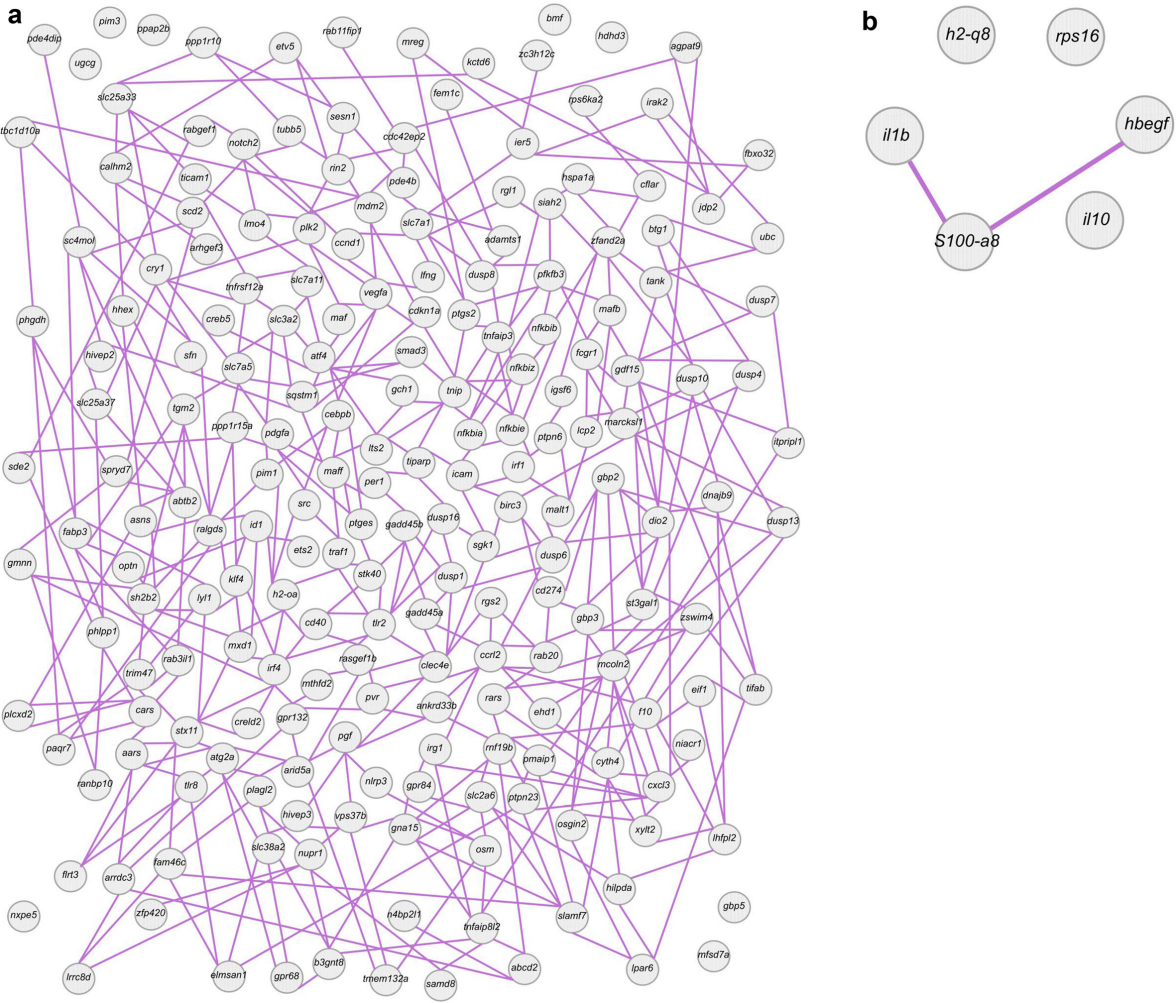
Gene network analysis of BALB/c and C57BL/6 macrophages infected with *La*-WT or *La*-arg^-^ for 4 h. (a) Network mapping based on co-expression analysis of the exclusively modulated genes based on the comparison of BALB/c_*La*-arg^-^ *vs.* BALB/c_*La*-WT. (b) Network mapping based on the co-expression analysis of the exclusively modulated genes for the comparison of C57BL/6_*La*-arg^-^ *vs.* C57BL/6_*La*-WT. *L. amazonensis* wild-type (*La*-WT) and *L. amazonensis* arginase knockout (*La*-arg^-^).

### Parasite arginase impacts the expression of genes related to host arginine transport and metabolism and the immune response

Since RNA-seq generates a large amount of data, we focused on the impact of parasite arginase on the host immune response and arginine transport and metabolism. The immune response of BALB/c-infected macrophages against *La*-WT was previously described by low levels of gene expression modulation that was related to limited immune response activation, specifically via chemokine signalling, the inflammatory response and susceptibility to infection [9]. In parallel, the immune response of C57BL/6-infected macrophages was described by high levels of gene expression modulation that was related to increased immune response activation, mainly via augmentation of recognition processes, activation of signalling cascades and moderate resistance to infection [9].

Among the list of 209 exclusively modulated genes in the comparison of BALB/c_*La*-arg^-^ *vs*. BALB/c_*La*-WT (Fig. 1c), 42 genes were related to the immune response processes, and most were upregulated (Fig. 1d, e) (Table S3). The transcripts related to the immune response processes included genes related to recognition, presentation and signalling (*cd40*, *cd274*, *clec4e*, *dusp1*, *dusp10*, *dusp13*, *dusp16*, *dusp4*, *dusp6*, *dusp7*, *dusp8*, *h2-oa*, *icam1*, irak2, *nlrp3*, *notch2*, *smad3*, *tank*, *ticam1*, *tifab*, *tlr2*, *tlr8* and *traf1*), transcription factors (*ier5*, *irf1*, *irf4*, *nfkbia*, *nfkbib*, *nfkbie* and *nfkbiz*), immunoregulatory molecules (*ccrl2*, *cxcl3*, *fcgr1*, *irg1*, *tnfaip3*, *tnfaip8l2*, *tnip1* and *tnfrsf12a*) and apoptosis (*gadd45a* and *gadd45b*) (Fig. 1e). These molecules involved in recognition, presentation and signalling play important roles and have implications for the susceptibility or resistance of cells to *Leishmania* infection [12, 47–50]. *Leishmania* infection triggered by Toll-like receptors (TLRs) promotes the activation of transcription factors and induces the production of pro-inflammatory cytokines [44, 46, 51, 52]. Apoptosis is another mechanism by which *Leishmania* evade the host immune response and survive inside the host [53].

Other modulated transcripts observed in the comparison of BALB/c_*La*-arg^-^ *vs.* BALB/c_*La*-WT are related to arginine transport and metabolism (Fig. 1d, e) (Table S3). Macrophages require exogenous arginine to proliferate and fulfil their metabolic needs. Therefore, the uptake of this amino acid may be a key regulatory step for physiological responses [54]. In this work, we observed the upregulation of *slc7a1*, which may indicate a host response to the absence of parasite arginase, since *slc7a1* encodes for the cationic amino acid transporter 1 (CAT1), which exhibits high affinity for arginine and has a major role in arginine homeostasis. In general, amino acid starvation in mammalian cells leads to increased CAT1 activity and arginine uptake [55, 56]. In addition, the absence of parasite arginase activity leads to reduced levels of ornithine and increased levels of l-arginine [57, 58]. The similarity between CAT1 and amino acid permease 3 (AAP3), an exclusively *Leishmania* amino acid transporter [2, 59–62], indicates that both may respond similarly to amino acid starvation [59].

*Slc3a2*, *solute carrier family 3 member 2*, is another exclusively modulated gene found in the comparison of BALB/c_*La*-arg^-^ *vs.* BALB/c_*La*-WT, indicating that its upregulation is another host response to the absence of parasite arginase activity. Previous studies have shown that the efflux of putrescine may be catalysed by the complex Slc3a2 and y+LAT through an arginine/diamine exchange mechanism. Interestingly, Slc3a2 can also interact with spermidine/spermine N-acetyltransferase [63]. Additionally, Slc3a2-deficient mice were not able to mount an efficient Th1 response against *L. major* infection due to low IFN-γ production [64].

The upregulation of these amino acid transporters (Fig. 1e) corroborates previous metabolomic profiling data showing increased levels of arginine and citrulline in BALB/c_*La*-arg^-^ compared to BALB/c_*La*-WT [57].

On the other hand, only six transcripts were identified among the list of the exclusively modulated genes in the comparison of C57BL/6_*La*-arg^-^ *vs.* C57BL/6_*La*-WT (Fig. 1c), and three of these genes were related to the immune response, one was related to growth factor (*hbegf*), one was related to calcium binding (*s100a8*) and one was related to ribosomal biogenesis (*rps16*) (Fig. 1d) (Table S4). Regarding the transcripts related to immune response processes, we observed the upregulation of the immunoregulatory molecules *h2-*q8, *il1b* and *il10* (Fig. 1e). Il1b and Il10 are cytokines previously described as important signalling factors during *Leishmania* infection, modulation of NO production and resistance to infection [65–67]. H2-Q8 is a molecule involved in antigen processing via MHC class I that can increase antigen presentation upon infection [68].

Network mapping based on co-expression analyses was performed and showed how the exclusively modulated genes interact with each other (Fig. 3a, b), revealing a much more complex co-expression structure for the comparison of BALB/c_*La*-arg^-^ *vs.* BALB/c_*La*-WT, including interaction of molecules in pathways related to immune response with those in pathways related to l-arginine transport and metabolism (Fig. 3a). In contrast, for the comparison of C57BL/6_*La*-arg^-^ *vs.* C57BL/6_*La*-WT we observed a weak co-expression network (Fig. 3b).

The modulated transcripts *f630028o10rik, e230016m11rik, gm129, gm10804*, *dnmt3aos* and *riken13* (Table S2) were not included in the gene network analysis comparing of BALB/c_*La*-arg^-^ *vs.* BALB/c_*La*-WT because they were not recognized by the GeneMANIA prediction server.

RT-qPCR validation assays were performed for four transcripts, and non-significant differences were observed for the RNA-seq data (Fig. S2).

Altogether, these data revealed that the parasite arginase activity has a high impact during the infection of BALB/c macrophages, mainly on transcripts related to the immune response, and arginine transport and metabolism.

### Parasite transcriptome profile reveals greater parasite gene expression modulation in BALB/c-infected macrophages than in C57BL/6-infected macrophages

RNA-seq data of the intracellular amastigote parasite form were also analysed according to the alignment with the closely related *L. mexicana* reference genome, since *L. amazonensis* has not been completely annotated [69–71]. The analysis identified 8282 transcripts (genome coverage of approximately 30 %).

**Fig. 4.**
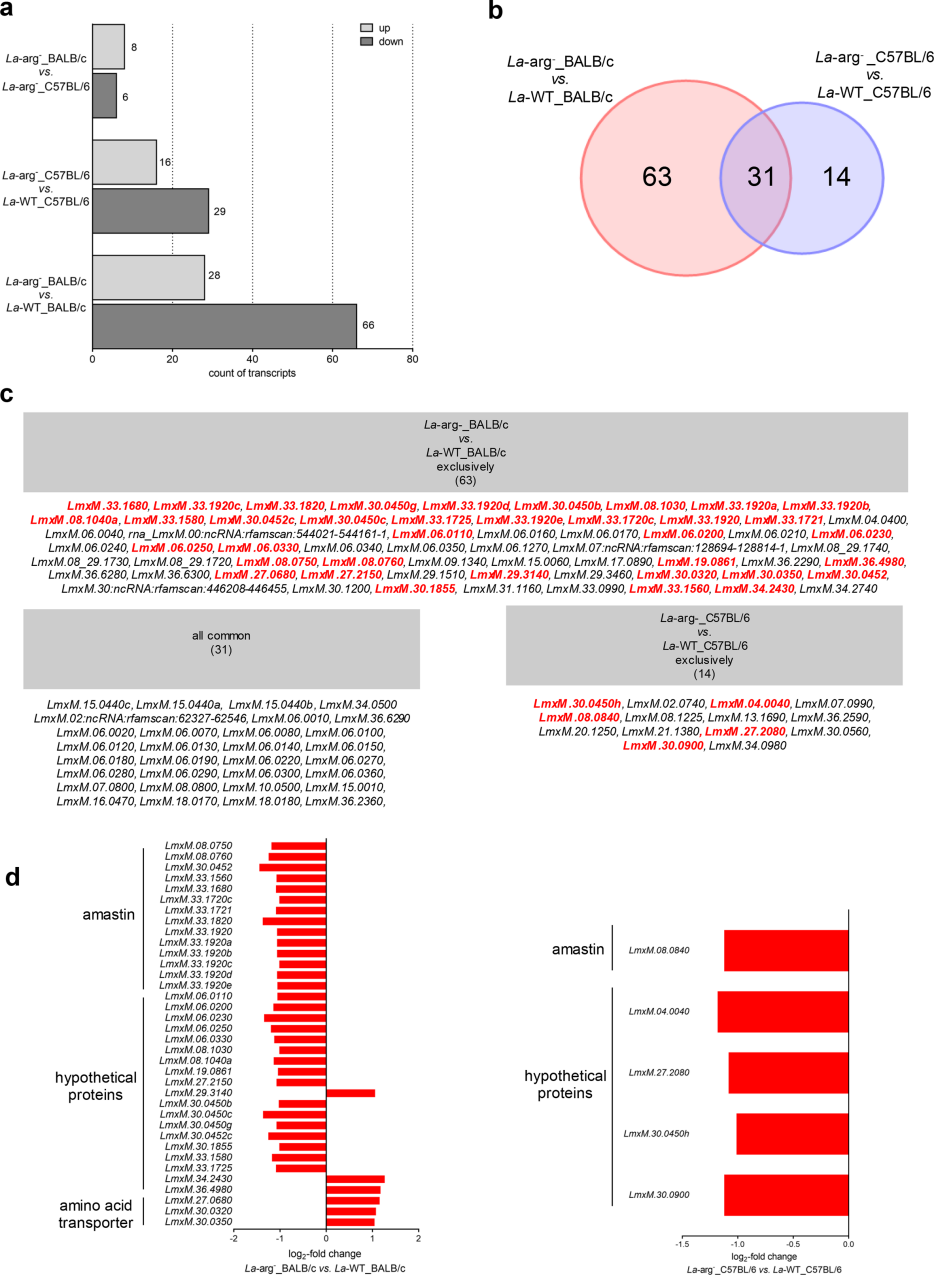
Functional analysis of the differentially expressed gene profile of *La*-WT and *La*-arg^-^ infecting BALB/c or C57BL/6 macrophages after 4 h. (a) Count of the differentially expressed genes (DEGs) upregulated (light grey) or downregulated (dark grey). (b) Venn diagram of the 108 DEGs in *La*-arg^-^_BALB/c *vs. La*-WT_BALB/c and *La*-arg^-^_C57BL/6 *vs. La*-WT_C57BL/6. (c) List of the exclusive and common transcripts for each comparison according to the Venn diagram analysis. (d) DEG profiles represented by log_2_-fold changes of the exclusively modulated genes in the comparisons of BALB/c_*La*-arg^-^ *vs.* BALB/c_*La*-WT and C57BL/6_*La*-arg^-^ *vs*. C57BL/6_*La*-WT. *L. amazonensis* wild-type (*La*-WT) and *L. amazonensis* arginase knockout (*La*-arg^-^). The data are from five independent biological replicates, considering fold change ≥2 and a *p*-value <0.05.

The DEG profile revealed 153 DEGs for the different parasite strain comparisons (Fig. 4a). As previously described, in the comparison of *La*-WT_BALB/c *vs. La*-WT_C57BL/6, we identified only one gene upregulated at the transcript level, a non-coding RNA (LmxM.32.ncRNA:rfamscan: 912 871–912 976), indicating that during early infection, the parasite does not significantly modulate its own gene expression, regardless of the genetic background of the murine host [9]. In contrast, the absence of parasite arginase triggered a differential gene expression modulation in both hosts. These differences may explain how the parasite is later able to replicate or subvert the mechanisms of defence depending on the host genetic background.

The essentiality of arginase in *Leishmania* was previously characterized in growth assays due to the requirement of putrescine supplementation [31], and *in vitro* and *in vivo* infections [21, 31, 32]. The role of parasite arginase was also related to the regulation of many genes involved in the uptake and metabolism of arginine and other amino acids [21, 28], as well as virulence factors [22].

For the comparison of *La*-arg^-^_BALB/c *vs. La*-arg^-^_C57BL/6, we identified six downregulated and eight upregulated transcripts. In contrast, we observed a greater gene expression modulation in the comparison of *La*-arg^-^- *vs. La*-WT-infected BALB/c macrophages, with 66 downregulated and 28 upregulated transcripts, compared to *La*-arg^-^- *vs. La*-WT-infected C57BL/6 macrophages, with 29 downregulated and 16 upregulated transcripts (Fig. 4a).

Considering the DEG profiles, we generated volcano plots comparing the fold changes in expression (log_2_) with the corresponding adjusted *p*-values (-log_10_) (Fig. 2c). We further analysed the volume plot (Fig. 2d) and identified the top five regulated transcripts for each comparison (Table 2).

**Table 2.** Top five differentially expressed genes in *La*-WT and *La*-arg^-^ intracellular amastigotes after 4 h of infection in BALB/c and C57BL/6 macrophages

ID	Product description	Fold change	*p*-value
*La*-arg-_BALB/c *vs. La*-WT_BALB/c
*LmxM.08_29.1730*	*histone h2a*, putative	0.38	2.06^−07^
*LmxM.08_29.1740*	*histone h2a*, putative	0.38	1.07^−07^
*LmxM.08_29.1720*	*histone h2a*, putative	0.38	2.06^−07^
*LmxM.06.0010*	*histone h4*	0.31	1.21^−05^
*LmxM.15.0010*	*histone h4*	0.30	2.21^−07^
	*La*-arg^-^_C57BL/6 *vs. La*-WT_C57BL/6		
*LmxM.15.0440b*	*unspecified product*	2.27	5.52^−05^
*LmxM.15.0440a*	*unspecified product*	2.26	1.46^−06^
*LmxM.30.0900*	*hypothetical protein*, conserved	0.49	9.51^−05^
*LmxM.15.0010*	*histone h4*	0.39	2.31^−05^
*LmxM.06.0010*	*histone h4*	0.39	5.60^−08^
	*La*-arg^-^_BALB/c *vs. La*-arg^-^_C57BL/6		
*LmxM.32.0316*	*heat-shock protein 83–1*	0.35	1.91^−07^
*LmxM.18.1370*	*heat-shock protein*, putative	0.41	5.60^−05^
*LmxM.28.2780*	*heat-shock protein hsp70*, putative	0.41	1.20^−05^
*LmxM.32.0314*	*heat-shock protein 83–1*	0.35	1.94^−07^
*LmxM.32.0312*	*heat-shock protein 83–1*	0.35	1.40^−07^

The top five most upregulated and downregulated genes among 8282transcripts previously defined as DEGs in the following comparisons: *La*-arg-_BALB/c *vs. La*-WT_BALB/c, *La*-arg-_C57BL/6 *vs. La*-WT_C57BL/6 and *La*-arg-_BALB/c *vs. La*-arg-_C57BL/6, based on the criteria of a foldchange≥2 and *p*-value ˂0.05. The list is based on the volume plot of differentially expressed genes.

The Venn diagram analysis revealed 63 exclusively modulated genes in the *La*-arg^-^_BALB/c compared to *La*-WT_BALB/c macrophages, 14 exclusively modulated genes in the *La*-arg^-^_C57BL/6 compared to *La*-WT_C57BL/6 macrophages and 31 commonly modulated genes (Fig. 4b.c).

### Parasite arginase activity impacts on the own parasite amino acid uptake

According to the transcriptome profile, we identified a greater gene expression modulation in the *La*-arg^-^_BALB/c *vs. La*-WT_BALB/c than in the *La*-arg^-^_C57BL/6 *vs. La*-WT_C57BL/6. Among the exclusively modulated genes identified in the *La*-arg^-^_BALB/c *vs. La*-WT_BALB/c, we identified 19 hypothetical proteins. In addition, we identified the upregulation of the three amino acids transporters (LmxM.27.0680, LmxM.30.0320 and LmxM.30.0350) (Fig. 4d) (Table S5), indicating that the gene expression of these transporters responds to the parasite arginase activity. LmxM.27.0680 is not characterized in *Leishmania*. LmxM.30.0320 and LmxM.0350 were previously described as DEGs in the transcriptome profiles of promastigotes and axenic amastigotes of *La*-arg^-^ and *La*-WT [21]. LmxM.30.0320 was also previously described as downregulated in *L. amazonensis* resistant to trivalent sodium stibogluconate compared to the susceptible parasite strain [72]. We also identified the modulation of 15 amastins, which were all downregulated (Fig. 4d and Table S5), indicating a mechanism by which parasite arginase can modulate amastigote survival and/or replication in BALB/c-infected macrophages.

In the comparison of *La*-arg^-^_C57BL/6 *vs. La*-WT_C57BL/6, we identified four downregulated hypothetical proteins and one downregulated amastin (Fig. 4d and Table S6) among the exclusively modulated genes, also indicating a mechanism by which the parasite arginase may modulate amastigote survival and/or replication, as was observed in BALB/c-infected macrophages. No transcript related to amino acid transport was identified.

Amastins are abundant surface antigens associated with the amastigote intracellular life stage and replication within the host cell [73–76]. The downregulation of amastin transcripts was previously demonstrated in the transcriptome profiles of promastigotes and axenic amastigotes from a *La*-arg^-^ and *La*-WT comparison, indicating that the parasite arginase activity impacts amastigote survival and replication [22]. The current work also revealed the downregulation of these amastins, mainly in the comparison of *La*-arg^-^_BALB/c *vs. La*-WT_BALB/c, corroborating our previous findings. RT-qPCR validation assays were performed for *amastin* (*LmxM.33.0960*) and parasite *arginase*, and non-significant differences were observed, confirming the RNA-seq data (Fig. S2).

The identification of several hypothetical proteins supports the importance of further studies to characterize these genes. The advent of RNA-seq has been shown to be an interesting approach for the identification of new important genes [28, 29]. The characterization of hypothetical protein-coding genes is continuously improving functional genomics annotations, revealing important biological features that enable a better understanding of signalling pathways, metabolism, stress response and drug resistance, which can potentially assist in the identification of new potential therapeutic targets [77–79].

In conclusion, we revealed the impact of parasite arginase on the modulation of the host immune response, and l-arginine uptake and metabolism, and showed that this modulation depends on the host genetic background determining the infection outcome.

## Supplementary Data

Supplementary material 1

Supplementary material 2
