# Supplementary material for: Dual transcriptome analysis reveals differential gene expression modulation influenced by Leishmania arginase and host genetic background
Source: Microb Genom. 2020 Sep 4;6(9):mgen000427. doi: 10.1099/mgen.0.000427 (PMC7643972; doi:10.1099/mgen.0.000427)
Supplement: Supplementary material 1 [file mgen-6-427-s001.pdf]

Dual transcriptome analysis reveals differential gene expression modulation influenced by *Leishmania* arginase and host genetic background

Juliana Ide Aoki[1*], Sandra Marcia Muxel[1], Maria Fernanda Laranjeira-Silva[1], Ricardo Andrade Zampieri[1], Karl Erik Müller[2,3], Audun Helge Nerland[2] and Lucile Maria Floeter-Winter[1*]

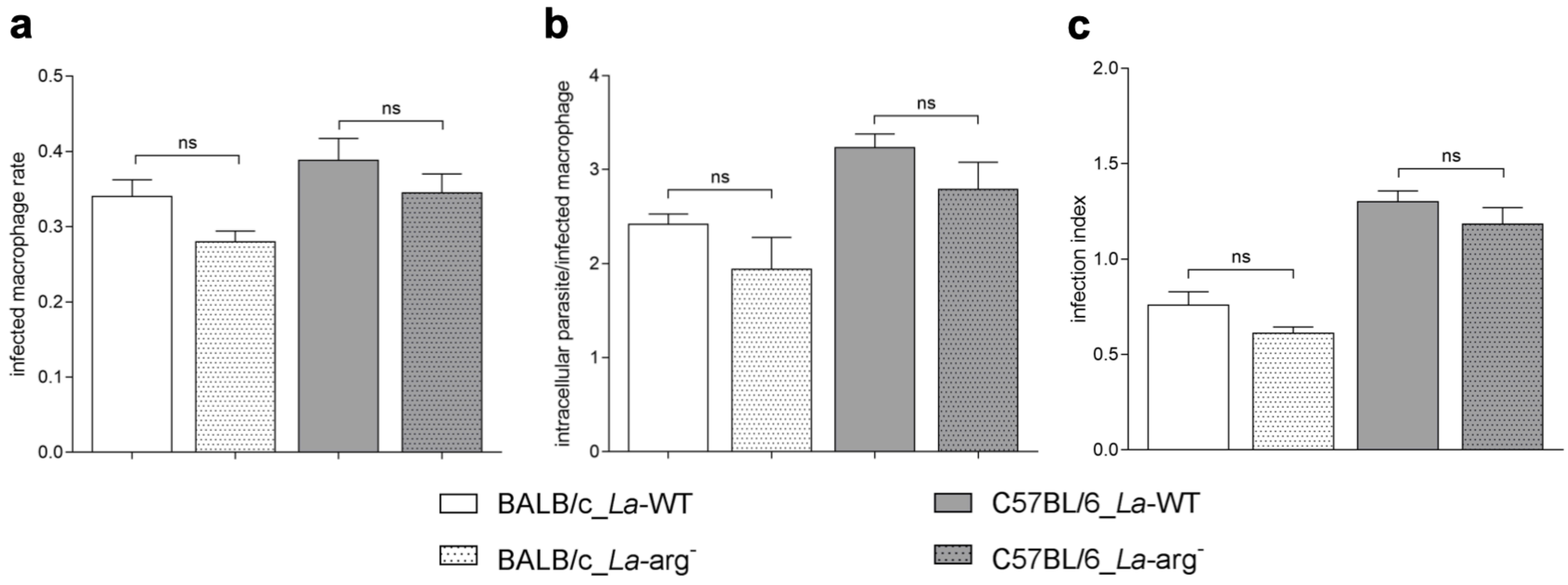

Fig. S1. Infection indexes of BALB/c and C57BL/6 mouse BMDMs infected with *La*-WT or *La*-arg^{-} for 4 h. BMDMs from BALB/c and C57BL/6 mice were infected with *La*-WT or *La*-arg^{-} (MOI 5:1) for 4 h. **(a)** Rate of infection was determined by the percentage of infected macrophages. **(b)** The number of intracellular parasite per infected macrophage. **(c)** The infection index was determined by the multiplication of the rate of infection and the mean number of intracellular parasites per infected macrophage. The bars indicate the mean ± SD of three independent biological replicates. No significant differences were observed for the comparisons of BALB/c_*La*-arg^{-} *vs.* BALB/c_*La*-WT and C57BL/6_*La*-arg^{-} *vs.* C57BL/6_*La*-WT. *L. amazonensis* wild type (*La*-WT). *L. amazonensis* arginase knockout (*La*-arg^{-}). ns, non-significant, considering p-value > 0.05.

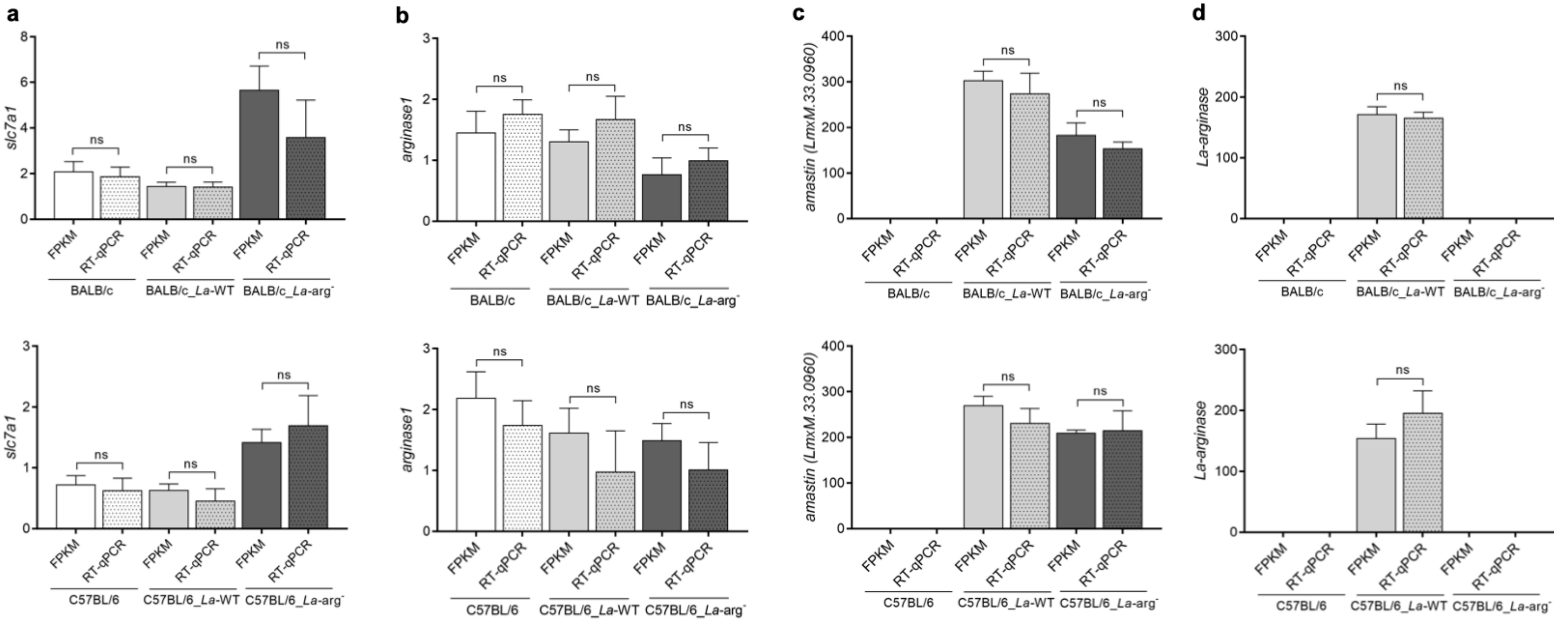

Fig. S2. RT-qPCR validation of 4 modulated transcripts in the comparison of BALB/c and C57BL/6 macrophages infected with *La*-WT or *La*-arg$^-$ for 4 h. (a) Comparative analysis of the expression levels of the host transcript *slc7a1* determined by FPKM and RT-qPCR. **(b)** Comparative analysis of the expression levels of the host transcript *arginase 1* determined by FPKM and RT-qPCR. **(c)** Comparative analysis of the expression levels of the parasite transcript amastin (*LmxM.33.0960*) determined by FPKM and RT-qPCR. **(d)** Comparative analysis of the expression levels of the parasite transcript *arginase* determined by FPKM and RT-qPCR. The RT-qPCR data is shown as the expression of the target genes normalized by the expression of *gapdh*. The bars represent the mean ± standard deviation of five independent biological replicates. *La*-WT, *L. amazonensis* wild type. *La*-arg$^-$, *L. amazonensis* arginase knockout. ns, non-significant for a comparison based on FPKM *vs.* RT-qPCR data.

Table S1. Oligonucleotides used for RT-qPCR validation

M. musculus primers	5´- 3´
*gapdh*_F	GGCAAATTCAACGGCACAGT
gapdh _R	CCTTTTGGCTCCACCCTTCA
*slc7a1*_F	CGTAATCGCCACTGTGACCT
*slc7a1*_R	GGCTGGTACCGTAAGACCAA
*arg1*_F	AGCACTGAGGAAAGCTGGTC
*arg1*_R	CAGACCGTGGGTTCTTCACA
L. amazonensis primers	5´- 3´
gapdh _F	TCAAGGTCGGTATCAACGGC
gapdh _R	TGCACCGTGTCGTACTTCAT
arg _F	TCCTGCACGACCTGAACATC
arg _R	CGCCATGGACACCACCTT
*amastin*_F	GGAGCGCTACTTCAGCTATGGA
*amastin*_R	CGGATCATCAATAAGACGATGTTG

Oligonucleotides sequences used in the RT-qPCR validation assays. *Mus musculus* primers: *glyceraldehyde 3-phosphate dehydrogenase* (*gapdh*), *solute carrier family 7 member 1* (*slc7a1*) and *arginase 1* (*arg1*). *Leishmania amazonensis* primers: *glyceraldehyde 3-phosphate dehydrogenase* (*gapdh*), *arginase* (*arg*) and *amastin* (LmxM.33.0820). Forward (F) and Reverse (R).

Table S3. List of the differentially expressed genes from the comparison of BALB/c_*La*-arg$^-$ *vs.* BALB/c_*La*-WT

ID	product description	fold change	*p*-value
slc3a2	*solute carrier family 3 (activators of dibasic and neutral amino acid transport), member 2*	2.66	7.91^{-08}
scd2	*stearoyl-Coenzyme A desaturase 2*	-2.26	7.27^{-06}
cd40	*CD40 antigen*	2.82	2.52^{-08}
cxcl3	*chemokine (C-X-C motif) ligand 3*	2.66	6.07^{-05}
rasgef1b	*RasGEF domain family, member 1B*	2.64	3.17^{-10}
zc3h12c	*zinc finger CCCH type containing 12C*	2.27	9.51^{-05}
marcksl1	*MARCKS-like 1*	2.25	1.30^{-07}
lmo4	*LIM domain only 4*	2.40	5.51^{-08}
tnip1	*TNFAIP3 interacting protein 1*	2.13	3.11^{-07}
malt1	*mucosa associated lymphoid tissue lymphoma translocation gene 1*	2.14	1.37^{-04}
nfkbiz	*nuclear factor of kappa light polypeptide gene enhancer in B cells inhibitor, zeta*	2.47	3.72^{-08}
elmsan1	*ELM2 and Myb/SANT-like domain containing 1*	2.08	4.11^{-06}
plcxd2	*phosphatidylinositol-specific phospholipase C, X domain containing 2*	2.94	8.27^{-07}
tnfrsf12a	*tumor necrosis factor receptor superfamily, member 12a*	2.08	3.76^{-05}
rnf19b	*ring finger protein 19B*	2.07	1.21^{-06}
.	*RIKEN cDNA E230013L22 gene*	2.21	1.84^{-06}
pfkfb3	*6-phosphofructo-2-kinase/fructose-2,6-biphosphatase 3*	2.08	3.41^{-05}
ptges	*prostaglandin E synthase*	2.69	5.70^{-07}
gbp3	*guanylate-binding protein 4*	2.08	9.59^{-05}
plk2	*polo-like kinase 2*	2.74	1.45^{-08}
st3gal1	*ST3 beta-galactoside alpha-2,3-sialyltransferase 1*	2.29	4.90^{-06}
sde2	*SDE2 telomere maintenance homolog (S. pombe)*	2.52	8.71^{-08}
h2-oa	*histocompatibility 2, O region alpha locus*	3.03	1.24^{-05}
ranbp10	*RAN binding protein 10*	2.01	5.57^{-03}
creb5	*cAMP responsive element binding protein 5*	2.00	5.80^{-05}
rab11fip1	*RAB11 family interacting protein 1 (class I)*	2.42	3.32^{-07}

aars	*alanyl-tRNA synthetase*	2.27	1.43^{-08}
gdf15	*growth differentiation factor 15*	7.44	1.93^{-07}
tnfsf14	*tumor necrosis factor (ligand) superfamily, member 14*	3.12	2.93^{-06}
dusp13	*dual specificity phosphatase 13*	2.79	6.93^{-06}
nupr1	*nuclear protein transcription regulator 1*	4.11	1.09^{-09}
jdp2	*Jun dimerization protein 2*	2.69	4.77^{-06}
rps6ka2	*ribosomal protein S6 kinase, polypeptide 2*	2.62	1.44^{-08}
arrdc3	*arrestin domain containing 3*	-2.69	2.11^{-05}
vps37b	*vacuolar protein sorting 37B (yeast)*	2.17	1.45^{-06}
chac1	*ChaC, cation transport regulator 1*	14.34	7.44^{-11}
spryd7	*SPRY domain containing 7*	2.06	6.14^{-07}
xylt2	*xylosyltransferase II*	-2.07	6.41^{-06}
lfng	*LFNG O-fucosylpeptide 3-beta-N-acetylglucosaminyltransferase*	-2.97	4.85^{-05}
slc25a37	*solute carrier family 25, member 37*	2.11	5.55^{-06}
vegfa	*vascular endothelial growth factor A*	2.46	9.39^{-09}
rab3il1	*RAB3A interacting protein (rabin3)-like 1*	-2.19	3.18^{-07}
rasd2	*RASD family, member 2*	3.09	6.20^{-06}
optn	*optineurin*	2.03	1.31^{-07}
irs2	*insulin receptor substrate 2*	2.79	7.31^{-07}
ets2	*E26 avian leukemia oncogene 2, 3' domain*	4.67	9.59^{-06}
hmgcs1	*hydroxymethylglutaryl-CoA synthase, cytoplasmic*	-2.27	4.84^{-06}
zfand2a	*zinc finger, AN1-type domain 2A*	2.04	7.74^{-09}
mafb	*v-maf musculoaponeurotic fibrosarcoma oncogene family, protein B (avian)*	-2.12	3.65^{-06}
hivep3	*human immunodeficiency virus type I enhancer binding protein 3*	2.33	7.20^{-06}
hilpda	*hypoxia inducible lipid droplet associated*	2.20	5.09^{-09}
f630028o10rik	*RIKEN cDNA F630028O10 gene*	-6.08	3.78^{-05}
ier5	*immediate early response 5*	2.01	9.00^{-07}
itpripl1	*inositol 1,4,5-triphosphate receptor interacting protein-like 1*	-2.59	1.61^{-07}
dusp16	*dual specificity phosphatase 16*	2.37	4.73^{-05}
fam46c	*family with sequence similarity 46, member C*	2.77	5.56^{-06}
tnfsf9	*tumor necrosis factor (ligand) superfamily, member 9*	2.08	9.81^{-05}

cebpb	*CCAAT/enhancer-binding protein beta isoform b*	2.44	5.35^{-06}
hivep2	*human immunodeficiency virus type I enhancer binding protein 2*	2.19	2.26^{-05}
tlr2	*toll-like receptor 2*	3.30	1.81^{-06}
nlrp3	*NLR family, pyrin domain containing 3*	3.16	6.21^{-05}
ccrl2	*C-C chemokine receptor-like 2*	6.05	1.20^{-13}
cdc42ep2	*CDC42 effector protein (Rho GTPase binding) 2*	2.05	1.05^{-05}
birc3	*baculoviral IAP repeat-containing 3*	2.22	3.74^{-08}
mfsd7a	*major facilitator superfamily domain containing 7A*	2.07	4.04^{-07}
osgin2	*oxidative stress induced growth inhibitor family member 2*	2.61	3.49^{-05}
gpr68	*G protein-coupled receptor 68*	2.16	1.21^{-06}
smad3	*SMAD family member 3*	2.34	8.52^{-06}
pim1	*proviral integration site 1*	2.36	2.98^{-07}
b3gnt8	*UDP-GlcNAc:betaGal beta-1,3-N-acetylglucosaminyltransferase 8*	-2.37	5.45^{-06}
paqr7	*progestin and adipoQ receptor family member VII*	-2.03	9.54^{-05}
creld2	*cysteine-rich with EGF-like domains 2*	2.00	3.53^{-07}
bmf	*BCL2 modifying factor*	-2.02	6.36^{-05}
notch2	*notch 2*	2.01	1.40^{-05}
irak2	*interleukin-1 receptor-associated kinase 2*	2.15	5.97^{-06}
klf4	*Kruppel-like factor 4 (gut)*	2.43	2.32^{-05}
cry1	*cryptochrome 1 (photolyase-like)*	2.50	1.90^{-07}
per1	*period circadian clock 1*	2.05	2.24^{-04}
slc38a2	*solute carrier family 38, member 2*	3.68	5.56^{-05}
stx11	*syntaxin 11*	3.27	1.60^{-06}
eif1	*eukaryotic translation initiation factor 1*	2.07	7.25^{-06}
abtb2	*ankyrin repeat and BTB (POZ) domain containing 2*	2.71	5.00^{-07}
ubc	*ubiquitin C*	2.15	2.22^{-05}
dusp8	*dual specificity phosphatase 8*	2.13	6.86^{-08}
sesn2	*sestrin 2*	5.83	7.47^{-09}
cd274	*CD274 antigen*	3.58	1.70^{-05}
cflar	*CASP8 and FADD-like apoptosis regulator isoform 4*	2.59	4.11^{-05}
n4bp2l1	*NEDD4 binding protein 2-like 1*	2.00	1.42^{-06}

hspa1a	*heat shock protein 1A*	2.44	$7,32^{-05}$
pim3	*proviral integration site 3*	2.46	$1,58^{-06}$
mdm2	*E3 ubiquitin-protein ligase Mdm2*	3.93	$1,24^{-05}$
slc7a11	*solute carrier family 7 (cationic amino acid transporter, y+ system), member 11*	2.92	5.62^{-05}
tnfaip3	*tumor necrosis factor, alpha-induced protein 3*	2.41	$1,09^{-05}$
ehd1	*EH-domain containing 1*	2.15	$5,83^{-07}$
bhlhe40	*basic helix-loop-helix family, member e40*	2.09	$2,61^{-06}$
dusp7	*dual specificity phosphatase 7*	-2.09	$1,15^{-05}$
gna15	*guanine nucleotide binding protein, alpha 15*	2.10	$6,18^{-07}$
dusp1	*dual specificity phosphatase 1*	5.98	$3,92^{-09}$
ptpn23	*protein tyrosine phosphatase, non-receptor type 23*	2.08	5.98^{-06}
herpud1	*homocysteine-inducible, endoplasmic reticulum stress-inducible, ubiquitin domain member 1*	8.08	$8,23^{-09}$
slc7a1	*high affinity cationic amino acid transporter 1*	3.18	4.10^{-05}
id1	*inhibitor of DNA binding 1*	-2.12	$6,45^{-05}$
lyl1	*lymphoblastomic leukemia 1*	-2.51	$1,69^{-06}$
tiparp	*TCDD-inducible poly(ADP-ribose) polymerase*	2.12	1.58^{-06}
fbxo32	*F-box protein 32*	2.06	7.42^{-07}
ankrd37	*ankyrin repeat domain 37*	-3.88	5.08^{-09}
fabp3	*fatty acid binding protein 3, muscle and heart*	2.08	3.60^{-08}
nfkbia	*nuclear factor of kappa light polypeptide gene enhancer in B cells inhibitor, alpha*	2.12	3.82^{-06}
nfkbie	*nuclear factor of kappa light polypeptide gene enhancer in B cells inhibitor, epsilon*	2.59	1.43^{-06}
nfkbib	*nuclear factor of kappa light polypeptide gene enhancer in B cells inhibitor, beta*	2.22	1.15^{-07}
tmem132a	*transmembrane protein 132A*	2.11	1.68^{-06}
pgf	*placental growth factor*	2.61	2.48^{-06}
adamts1	*a disintegrin-like and metallopeptidase (reprolysin type) with thrombospondin type 1 motif, 1*	2.37	1.06^{-05}
pde4dip	*myomegalin isoform 6*	2.61	1.96^{-06}
zfp420	*zinc finger protein 420*	2.04	1.51^{-05}
slc7a5	*solute carrier family 7 (cationic amino acid transporter, y+ system), member 5*	3.68	3.40^{-08}
lpar6	*lysophosphatidic acid receptor 6*	-2.41	1.08^{-05}
phlpp1	*PH domain and leucine rich repeat protein phosphatase 1*	2.24	9.85^{-07}
sh2b2	*SH2B adapter protein 2 isoform 2*	2.72	1.59^{-07}

abcd2	*ATP-binding cassette, sub-family D (ALD), member 2*	-2.15	4.89^{-06}
cxcl2	*chemokine (C-X-C motif) ligand 2*	8.60	1.20^{-09}
ptpn6	*protein tyrosine phosphatase, non-receptor type 6*	-2.05	4.52^{-05}
ptgs2	*prostaglandin-endoperoxide synthase 2*	2.14	3.09^{-05}
gpr84	*G protein-coupled receptor 84*	2.19	1.61^{-05}
tnfaip8l2	*tumor necrosis factor, alpha-induced protein 8-like 2*	-2.31	2.90^{-05}
tank	*TRAF family member-associated Nf-kappa B activator*	2.06	3.56^{-06}
ppap2b	*phosphatidic acid phosphatase type 2B*	2.64	1.60^{-08}
slc2a6	*solute carrier family 2 (facilitated glucose transporter), member 6*	2.03	1.51^{-06}
ddit3	*DNA damage-inducible transcript 3 protein*	5.41	1.30^{-06}
gadd45a	*growth arrest and DNA-damage-inducible 45 alpha*	4.21	3.18^{-07}
sgk1	*serum/glucocorticoid regulated kinase 1*	2.77	1.99^{-09}
gch1	*GTP cyclohydrolase 1*	2.19	1.01^{-05}
igsf6	*immunoglobulin superfamily, member 6*	2.32	9.15^{-06}
kctd6	*potassium channel tetramerisation domain containing 6*	2.24	2.20^{-05}
pmaip1	*phorbol-12-myristate-13-acetate-induced protein 1*	2.20	7.46^{-06}
plagl2	*pleiomorphic adenoma gene-like 2*	2.29	5.33^{-05}
mcoln2	*mucolipin 2*	2.07	9.65^{-08}
il1a	*interleukin 1 alpha*	2.41	1.71^{-03}
trim47	*tripartite motif-containing 47*	-2.01	6.93^{-06}
fem1c	*fem-1 homolog c (C.elegans)*	2.45	1.76^{-05}
ifrd1	*interferon-related developmental regulator 1*	6.02	9.83^{-08}
zbtb21	*zinc finger and BTB domain containing 21*	2.27	2.10^{-08}
ugcg	*UDP-glucose ceramide glucosyltransferase*	2.05	4.25^{-05}
rabgef1	*RAB guanine nucleotide exchange factor (GEF) 1*	2.29	4.30^{-07}
gmnn	*geminin*	-2.07	2.95^{-07}
rgl1	*ral guanine nucleotide dissociation stimulator,-like 1*	2.32	1.21^{-06}
ticam1	*toll-like receptor adaptor molecule 1*	2.26	5.19^{-06}
pvr	*poliovirus receptor*	2.48	3.24^{-09}
pde4b	*phosphodiesterase 4B, cAMP specific*	3.04	3.28^{-10}
maff	*v-maf musculoaponeurotic fibrosarcoma oncogene family, protein F (avian)*	3.92	7.62^{-08}

maf	*avian musculoaponeurotic fibrosarcoma (v-maf) AS42 oncogene homolog*	-2.58	9.71^{-07}
cars	*cysteinyl-tRNA synthetase*	2.36	1.98^{-06}
clec4e	*C-type lectin domain family 4, member e*	4.21	5.77^{-07}
slc25a33	*solute carrier family 25, member 33*	2.33	4.84^{-05}
arg2	*arginase type II*	2.45	2.40^{-09}
stk40	*serine/threonine kinase 40*	2.00	1.30^{-05}
rars	*arginyl-tRNA synthetase*	2.10	3.53^{-06}
gpr132	*G protein-coupled receptor 132*	2.29	1.34^{-07}
rgs2	*regulator of G-protein signaling 2*	-2.00	1.21^{-06}
ralgds	*ral guanine nucleotide dissociation stimulator*	2.88	2.10^{-09}
rab20	*RAB20, member RAS oncogene family*	2.68	4.04^{-07}
mthfd2	*methylenetetrahydrofolate dehydrogenase (NAD+dependent)*	3.68	3.80^{-09}
f10	*coagulation factor X*	2.48	1.02^{-07}
atg2a	*autophagy related 2A*	2.05	1.10^{-05}
etv5	*ets variant gene 5*	-2.00	6.83^{-06}
gem	*GTP binding protein (gene overexpressed in skeletal muscle)*	6.01	2.46^{-08}
trib3	*tribbles homolog 3 (Drosophila)*	8.64	2.61^{-10}
hdhd3	*haloacid dehalogenase-like hydrolase domain containing 3*	-2.07	2.14^{-05}
tbc1d10a	*TBC1 domain family, member 10a*	2.27	4.18^{-06}
gbp5	*guanylate binding protein 5*	2.55	3.86^{-05}
btg1	*B cell translocation gene 1, anti-proliferative*	2.48	8.12^{-05}
rin2	*Ras and Rab interactor 2*	-2.87	7.92^{-08}
e230016m11rik	*RIKEN cDNA E230016M11 gene*	2.34	5.53^{-08}
ccnd1	*cyclin D1*	-2.19	2.85^{-06}
sc4mol	*sterol-C4-methyl oxidase-like*	-2.00	2.52^{-08}
hspa5	*heat shock protein 5*	3.14	6.25^{-08}
cxcl1	*chemokine (C-X-C motif) ligand 1*	7.03	2.31^{-08}
tubb5	*tubulin, beta 5 class I*	-2.04	2.56^{-09}
phgdh	*3-phosphoglycerate dehydrogenase*	4.24	5.92^{-06}
rgs1	*regulator of G-protein signaling 1*	2.49	1.39^{-06}
ern1	*endoplasmic reticulum (ER) to nucleus signalling 1*	3.73	1.04^{-05}

slamf7	*SLAM family member 7*	2.34	1.81^{-05}
ppp1r10	*protein phosphatase 1, regulatory subunit 10*	2.05	6.29^{-08}
tgm2	*transglutaminase 2, C polypeptide*	2.52	2.09^{-08}
tlr8	*toll-like receptor 8*	-2.43	1.07^{-07}
icosl	*icos ligand*	2.44	5.29^{-08}
atf4	*activating transcription factor 4*	4.06	3.04^{-08}
irf1	*interferon regulatory factor 1*	2.29	1.17^{-06}
irg1	*immunoresponsive gene 1*	3.75	2.06^{-10}
irf4	*interferon regulatory factor 4*	5.57	2.67^{-11}
lhfpl2	*lipoma HMGIC fusion partner-like 2*	2.09	3.06^{-05}
tifab	*TRAF-interacting protein with forkhead-associated domain, family member B*	-3.08	3.86^{-06}
dusp6	*dual specificity phosphatase 6*	-5.62	1.74^{-07}
agpat9	*1-acylglycerol-3-phosphate O-acyltransferase 9*	2.75	1.70^{-07}
gm129	*predicted gene 129*	2.34	1.81^{-05}
fcgr1	*Fc receptor, IgG, high affinity I*	-2.10	5.24^{-08}
arhgef3	*rho guanine nucleotide exchange factor 3 isoform 3*	2.86	4.03^{-09}
ppp1r15a	*protein phosphatase 1, regulatory (inhibitor) subunit 15A*	4.68	1.06^{-09}
gadd45b	*growth arrest and DNA-damage-inducible 45 beta*	2.03	3.54^{-08}
cdkn1a	*cyclin-dependent kinase inhibitor 1A (P21)*	2.01	1.85^{-06}
dusp10	*dual specificity phosphatase 10*	4.22	5.88^{-07}
dnajb9	*DnaJ (Hsp40) homolog, subfamily B, member 9*	2.32	1.91^{-05}
pdgfa	*platelet derived growth factor, alpha*	2.05	2.67^{-06}
traf1	*TNF receptor-associated factor 1*	3.47	5.51^{-07}
gbp2	*guanylate binding protein 2*	2.25	1.95^{-05}
calhm2	*calcium homeostasis modulator 2*	-2.16	1.94^{-05}
gm10804	*predicted gene 10804*	2.14	2.47^{-05}
dnmt3aos	*DNA methyltransferase 3A, opposite strand*	2.62	1.43^{-07}
dusp4	*dual specificity phosphatase 4*	2.81	5.44^{-08}
asns	*asparagine synthetase*	3.48	1.16^{-09}
niacr1	*niacin receptor 1*	3.90	1.07^{-07}
mxd1	*MAX dimerization protein 1*	2.05	2.88^{-07}

arid5a	*AT-rich interactive domain-containing protein 5A isoform 6*	2.62	4.33^{-07}
sfn	*stratifin*	2.22	5.12^{-06}
dio2	*deiodinase, iodothyronine, type II*	2.23	1.81^{-06}
mreg	*melanoregulin*	2.62	5.28^{-08}
siah2	*seven in absentia 2*	2.50	6.76^{-07}
csrnp1	*cysteine-serine-rich nuclear protein 1*	2.94	2.43^{-08}
zswim4	*zinc finger SWIM-type containing 4*	2.48	3.18^{-07}
lcp2	*lymphocyte cytosolic protein 2*	2.63	1.08^{-08}
manf	*mesencephalic astrocyte-derived neurotrophic factor*	2.26	6.81^{-09}
osm	*oncostatin M*	4.74	5.42^{-09}
sqstm1	*sequestosome-1 isoform 2*	2.41	2.65^{-09}
ankrd33b	*ankyrin repeat domain 33B*	2.65	1.30^{-07}
samd8	*sterile alpha motif domain containing 8*	2.23	1.44^{-06}
flrt3	*fibronectin leucine rich transmembrane protein 3*	2.09	1.12^{-06}
lrrc8d	*leucine rich repeat containing 8D*	2.32	9.41^{-05}
cyth4	*cytohesin 4*	-2.29	6.32^{-07}
sesn1	*sestrin 1*	-2.39	1.36^{-09}
hhex	*hematopoietically expressed homeobox*	-2.49	9.42^{-07}
src	*Rous sarcoma oncogene*	2.16	1.35^{-05}
nxpe5	*neurexophilin and PC-esterase domain family, member 5*	-2.25	6.24^{-06}
icam1	*intercellular adhesion molecule 1*	3.30	8.35^{-09}

List of the 233 DEGs from the comparison of BALB/c_*La*-arg$^-$ *vs.* BALB/c_*La*-WT, considering a fold change ≥ 2 and *p*-value < 0.05. *L. amazonensis* wild-type (*La*-WT) and *L. amazonensis* arginase knockout (*La*-arg$^-$).

Table S4. List of the differentially expressed genes from the comparison of C57BL/6_*La*-arg^{-} *vs.* C57BL/6_*La*-WT

ID	product description	fold change	*p*-value
tnfsf14	*tumor necrosis factor (ligand), member 14*	2.09	1.69^{-05}
chac1	*ChaC, cation transport regulator 1*	3.02	2.28^{-05}
rasd2	*RASD family, member 2*	2.20	3.16^{-08}
irs2	*insulin receptor substrate 2*	2.09	1.00^{-07}
hmgcs1	*hydroxymethylglutaryl-CoA synthase, cytoplasmic*	-2.04	1.37^{-07}
tnfsf9	*tumor necrosis factor (ligand), member 9*	2.07	4.53^{-05}
il1b	*interleukin 1 beta*	2.01	1.23^{-05}
sesn2	*sestrin 2*	2.13	2.44^{-07}
bhlhe40	*basic helix-loop-helix family, member e40*	2.06	5.85^{-07}
herpud1	*homocysteine-inducible, ER stress-inducible, ubiquitin-like domain member 1*	3.78	1.04^{-09}
ankrd37	*ankyrin repeat domain 37*	-3.02	3.30^{-07}
hbegf	*heparin-binding EGF-like growth factor*	2.12	1.17^{-06}
cxcl2	*chemokine (C-X-C motif) ligand 2*	2.25	1.29^{-05}
ddit3	*DNA damage-inducible transcript 3 protein*	3.29	3.11^{-06}
rps16	*ribosomal protein S16*	2.13	9.07^{-06}
il1a	*interleukin 1 alpha*	3.11	6.64^{-06}
ifrd1	*interferon-related developmental regulator 1*	2.07	3.66^{-08}
arg2	*arginase type II*	2.13	3.51^{-05}
h2-q8	*histocompatibility 2, Q region locus 8*	2.37	1.97^{-07}
gem	*GTP binding protein*	2.45	1.51^{-05}
trib3	*tribbles homolog 3 (Drosophila)*	2.24	4.62^{-06}
hspa5	*heat shock protein 5*	2.68	2.55^{-09}
cxcl1	*chemokine (C-X-C motif) ligand 1*	2.36	4.53^{-05}
rgs1	*regulator of G-protein signaling 1*	2.26	1.57^{-04}
ern1	*ER to nucleus signalling 1*	2.04	1.68^{-05}
icosl	*icos ligand*	2.06	1.96^{-06}

il10	*interleukin 10*	2.31	4.31^{-05}
s100a8	*S100 calcium binding protein A8*	-2.23	7.46^{-06}
csrnp1	*cysteine-serine-rich nuclear protein 1*	2.05	8.69^{-05}
manf	*mesencephalic astrocyte-derived neurotrophic factor*	2.08	5.40^{-07}

List of the 30 DEGs from the comparison of C57BL/6_*La*-arg^{-} *vs.* C57BL/6_*La*-WT, considering a fold change ≥ 2 and *p*-value < 0.05. *L. amazonensis* wild-type (*La*-WT) and *L. amazonensis* arginase knockout (*La*-arg^{-}).

Table S5. List of the differentially expressed genes from the comparison of *La*-arg^{-}_BALB/c *vs. La*-WT_BALB/c

ID	product description	fold change	*p*-value
LmxM.33.1680	*amastin-like surface protein, putative*	-2.11	2.51^{-07}
LmxM.33.1920c	*amastin-like surface protein, putative (fragment)*	-2.01	3.74^{-05}
LmxM.33.1820	*amastin-like surface protein, putative*	-2.59	2.92^{-05}
LmxM.30.0450g	*hypothetical protein*	-2.10	8.88^{-06}
LmxM.33.1920d	*amastin-like surface protein, putative*	-2.09	$4,58^{-05}$
LmxM.15.0440c	*hypothetical protein (fragment)*	2.32	3.25^{-06}
LmxM.30.0450b	*hypothetical protein*	-2.02	6.79^{-04}
LmxM.08.1030	*hypothetical protein*	-2.02	3.84^{-05}
LmxM.33.1920a	*amastin-like surface protein, putative*	-2.09	4.19^{-05}
LmxM.33.1920b	*amastin-like surface protein, putative*	-2.09	2.32^{-05}
LmxM.08.1040a	*hypothetical protein*	-2.20	1.03^{-07}
LmxM.33.1580	*hypothetical protein*	-2.25	3.65^{-06}
LmxM.30.0452c	*hypothetical protein*	-2.37	1.57^{-05}
LmxM.30.0450c	*hypothetical protein*	-2.40	4.47^{-06}
LmxM.33.1725	*hypothetical protein*	-2.12	2.16^{-07}
LmxM.15.0440a	*hypothetical protein*	2.07	1.50^{-07}
LmxM.33.1920e	*amastin-like surface protein, putative*	-2.07	5.82^{-06}
LmxM.33.1720c	*amastin-like surface protein, putative*	-2.02	6.93^{-05}
LmxM.15.0440b	*hypothetical protein*	2.14	1.22^{-06}
LmxM.33.1920	*amastin-like surface protein, putative*	-2.09	$1,13^{-07}$
LmxM.33.1721	*amastin-like surface protein, putative*	-2.12	$1,92^{-05}$
LmxM.00:ncRNA	*ncRNA:rfamscan:544021-544161-1*	2.54	1.71^{-07}
LmxM.02:ncRNA	*unspecified product: 62327-62546*	2.65	1.12^{-07}
LmxM.04.0400	*anaphase promoting complex, subunit 10-like protein*	-2.34	4.58^{-05}
LmxM.06.0010	*histone H4*	-2.78	1.21^{-05}
LmxM.06.0020	*hypothetical protein, conserved*	-2.21	2.36^{-05}
LmxM.06.0040	*hypothetical protein, conserved*	-2.36	1.75^{-06}
LmxM.06.0070	*hypothetical protein, conserved*	-2.62	1.20^{-05}

LmxM.06.0080	*ATP-binding cassette protein subfamily G, member 1, putative (ABCG1)*	-2.18	3.11^{-05}
LmxM.06.0100	*ATP-binding cassette protein subfamily G, member 3, putative (ABCG3)*	-2.14	1.38^{-05}
LmxM.06.0110	*hypothetical protein, conserved*	-2.07	1.10^{-05}
LmxM.06.0120	*cyclophilin*	-2.55	1.75^{-05}
LmxM.06.0130	*hypothetical protein, conserved*	-2.94	4.44^{-06}
LmxM.06.0140	*proteasome beta 6 subunit, putative*	-2.29	2.41^{-05}
LmxM.06.0150	*hypothetical protein, conserved*	-2.42	2.47^{-07}
LmxM.06.0160	*hypothetical protein, conserved*	-2.31	6.31^{-05}
LmxM.06.0170	*hypothetical protein, conserved*	-2.13	1.44^{-07}
LmxM.06.0180	*kinesin-like protein*	-2.57	5.92^{-06}
LmxM.06.0190	*hypothetical protein, conserved*	-2.43	1.08^{-07}
LmxM.06.0200	*hypothetical protein, conserved*	-2.21	7.43^{-06}
LmxM.06.0210	*hypothetical protein, conserved*	-2.18	4.03^{-06}
LmxM.06.0220	*hypothetical protein, conserved*	-2.69	1.99^{-05}
LmxM.06.0230	*hypothetical protein, conserved*	-2.54	2.39^{-05}
LmxM.06.0240	*present in the outer mitochondrial membrane proteome 34 (POMP34)*	-2.10	3.86^{-05}
LmxM.06.0250	*hypothetical protein, conserved*	-2.28	2.04^{-06}
LmxM.06.0270	*hypothetical protein, conserved*	-2.56	1.19^{-06}
LmxM.06.0280	*hypothetical protein, conserved*	-2.26	1.93^{-06}
LmxM.06.0290	*ribonuclease H1, putative*	-2,36	3.64^{-06}
LmxM.06.0300	*MYND finger domain-like protein*	-2.48	5.38^{-05}
LmxM.06.0330	*hypothetical protein, conserved*	-2.17	6.08^{-06}
LmxM.06.0340	*oligopeptidase B-like protein*	-2.13	2.08^{-06}
LmxM.06.0350	*NAD(P)-dependent steroid dehydrogenase protein, putative*	-3.00	1.91^{-05}
LmxM.06.0360	*hypothetical protein, conserved*	-2.30	4.32^{-07}
LmxM.06.1270	*coproporphyrinogen III oxidase, putative*	2.14	1.01^{-07}
LmxM.07:ncRNA	*ncRNA:rfamscan:128694-128814-1*	62.95	1.78^{-07}
LmxM.07.0800	*flavoprotein subunit-like protein*	2.34	2.18^{-05}
LmxM.08_29.1740	*histone H2A, putative*	-2.32	1.07^{-07}
LmxM.08_29.1730	*histone H2A, putative*	-2.29	2.06^{-07}
LmxM.08_29.1720	*histone H2A, putative*	-2.29	2.06^{-07}

LmxM.08.0800	*amastin-like protein, putative*	-2.48	1.42^{-05}
LmxM.08.0750	*amastin-like protein, putative*	-2.27	1.89^{-07}
LmxM.08.0760	*amastin-like protein, putative*	-2.37	1.39^{-05}
LmxM.09.1340	*histone H2B*	-2.59	$1,37^{-05}$
LmxM.10.0500	*hypothetical protein, conserved*	2.16	4.99^{-05}
LmxM.15.0010	*histone H4*	-2.93	2.21^{-07}
LmxM.15.0060	*eukaryotic translation initiation factor 4 gamma type 1, putative (eif4g1)*	2.16	4.54^{-07}
LmxM.16.0470	*hypothetical protein, conserved*	-2.06	1.50^{-07}
LmxM.17.0890	*hypothetical protein, conserved*	-2.00	3.90^{-05}
LmxM.18.0170	*hypothetical protein, unknown function*	2.31	3.47^{-07}
LmxM.18.0180	*hypothetical protein, unknown function*	2.43	1.23^{-07}
LmxM.19.0861	*hypothetical protein*	-2.06	2.11^{-07}
LmxM.36.2290	*protein kinase, putative*	2.83	4.35^{-07}
LmxM.36.2360	*tyrosine aminotransferase, putative*	2.32	3.34^{-05}
LmxM.36.4980	*hypothetical protein, conserved*	2.26	2.93^{-06}
LmxM.36.6280	*glucose transporter 3 (GT3)*	2.03	5.70^{-05}
LmxM.36.6290	*glucose transporter 2 (GT2)*	2.32	3.04^{-06}
LmxM.36.6300	*glucose transporter 1 (GT1)*	2.16	5.24^{-07}
LmxM.27.0680	*amino acid permease, putative*	2.22	8.89^{-05}
LmxM.27.2150	*hypothetical protein, unknown function*	-2.09	1.26^{-05}
LmxM.29.1510	*p1/s1 nuclease*	-2.49	2.59^{-07}
LmxM.29.3140	*hypothetical protein, conserved*	2.07	1.98^{-07}
LmxM.29.3460	*zinc-binding protein (Yippee), putative*	-2.01	1.69^{-06}
LmxM.30.0320	*amino acid transporter, putative*	2.10	9.38^{-05}
LmxM.30.0350	*amino acid transporter aATP11, putative*	2.07	4.43^{-07}
LmxM.30.0452	*amastin, putative*	-2.72	1.10^{-06}
LmxM.30:ncRNA	*unspecified product: 446208-446455*	-2.16	8.79^{-06}
LmxM.30.1200	*hypothetical protein, unknown function*	2.06	5.25^{-06}
LmxM.30.1855	*hypothetical protein*	-2.02	1.57^{-06}
LmxM.31.1160	*long chain polyunsaturated fatty acid elongation enzyme-like protein*	2.20	1.34^{-07}
LmxM.33.0990	*p-glycoprotein*	2.38	4.51^{-07}

LmxM.33.1560	*amastin-like surface protein, putative*	-2.11	5.50^{-05}
LmxM.34.0500	*proteophosphoglycan ppg3, putative*	2.43	2.85^{-07}
LmxM.34.2430	*hypothetical protein, conserved*	2.40	1.83^{-07}
LmxM.34.2740	*galactokinase-like protein*	2.57	1.96^{-07}

List of the 94 DEGs from the comparison of *La*-arg^{-}_BALB/c *vs. La*-WT_BALB/c, considering a fold change ≥ 2 and *p*-value < 0.05. *L. amazonensis* wild-type (*La*-WT) and *L. amazonensis* arginase knockout (*La*-arg^{-}).

Table S6. List of the differentially expressed genes from the comparison of *La*-arg^{-}_BALB/c *vs.* *La-WT*_BALB/c

ID	product description	fold change	*p*-value
LmxM.15.0440c	*hypothetical protein (fragment)*	2.69	5.04^{-05}
LmxM.15.0440a	*hypothetical protein*	2.48	1.46^{-05}
LmxM.30.0450h	*hypothetical protein*	-2.01	2.43^{-06}
LmxM.15.0440b	*hypothetical protein*	2.61	5.52^{-05}
LmxM.02:ncRNA	*unspecified product: 62327-62546*	2.48	1.12^{-06}
LmxM.02.0740	*peptidyl dipeptidase, putative*	2.13	8.98^{-07}
LmxM.04.0040	*hypothetical protein*	-2.26	1.93^{-06}
LmxM.06.0010	*histone H4*	-2.48	5.60^{-08}
LmxM.06.0020	*hypothetical protein, conserved*	-2.67	2.51^{-05}
LmxM.06.0070	*hypothetical protein, conserved*	-2.26	1.29^{-05}
LmxM.06.0080	*ATP-binding cassette protein subfamily G, member 1, putative (ABCG1)*	-2.03	1.16^{-05}
LmxM.06.0100	*ATP-binding cassette protein subfamily G, member 3, putative (ABCG3)*	-2.09	3.43^{-06}
LmxM.06.0120	*cyclophilin*	-2.21	3.68^{-06}
LmxM.06.0130	*hypothetical protein, conserved*	-2.38	4.63^{-05}
LmxM.06.0140	*proteasome beta 6 subunit, putative*	-2.10	7.25^{-06}
LmxM.06.0150	*hypothetical protein, conserved*	-2.55	3.01^{-05}
LmxM.06.0180	*kinesin-like protein*	-2.04	5.66^{-07}
LmxM.06.0190	*hypothetical protein, conserved*	-2.02	2.38^{-05}
LmxM.06.0220	*hypothetical protein, conserved*	-2.50	1.22^{-07}
LmxM.06.0270	*hypothetical protein, conserved*	-2.73	1.34^{-05}
LmxM.06.0280	*hypothetical protein, conserved*	-2.03	3.77^{-09}
LmxM.06.0290	*ribonuclease H1, putative*	-2.08	7.99^{-06}
LmxM.06.0300	*MYND finger domain-like protein*	-2.48	1.28^{-09}
LmxM.06.0360	*hypothetical protein, conserved*	-2.00	2.79^{-07}
LmxM.07.0800	*flavoprotein subunit-like protein*	2.66	3.00^{-07}
LmxM.07.0990	*nucleolar RNA-binding protein, putative*	-2.05	2.28^{-06}
LmxM.08.0800	*amastin-like protein, putative*	-2.11	1.77^{-05}

LmxM.08.0840	*amastin-like protein, putative*	-2.17	1.56^{-05}
LmxM.08.1225	*hypothetical protein, unknown function*	-2.38	4.95^{-05}
LmxM.10.0500	*hypothetical protein, conserved*	2.16	1.43^{-05}
LmxM.13.1690	*protein associated with differentiation*	2.00	3.89^{-07}
LmxM.15.0010	*histone H4*	-2.35	2.31^{-07}
LmxM.16.0470	*hypothetical protein, conserved*	-2.62	1.43^{-06}
LmxM.18.0170	*hypothetical protein, unknown function*	2.23	3.05^{-05}
LmxM.18.0180	*hypothetical protein, unknown function*	2.06	3.82^{-05}
LmxM.36.2360	*tyrosine aminotransferase, putative*	2.08	3.59^{-06}
LmxM.36.2590	*membrane-bound acid phosphatase 2*	2.06	8.27^{-07}
LmxM.36.6290	*glucose transporter 2 (GT2)*	2.18	4.78^{-08}
LmxM.20.1250	*hypothetical protein, conserved*	-2.14	8.70^{-06}
LmxM.21.1380	*hypothetical protein, conserved*	2.21	1.08^{-06}
LmxM.27.2080	*hypothetical protein, unknown function*	-2.12	4.37^{-07}
LmxM.30.0560	*mevalonate kinase, putative*	-2.14	1.20^{-06}
LmxM.30.0900	*hypothetical protein, conserved*	-2.17	9.51^{-05}
LmxM.34.0500	*proteophosphoglycan ppg3, putative*	2.31	1.16^{-05}
LmxM.34.0980	*aldose 1-epimerase-like protein*	2.31	1.55^{-07}

List of the 45 DEGs from the comparison of *La*-arg$^-$_C57BL/6 *vs. La*-WT_C57BL/6, considering a fold change ≥ 2 and *p*-value < 0.05. *L. amazonensis* wild-type (*La*-WT) and *L. amazonensis* arginase knockout (*La*-arg$^-$).
